# Perspectives of Parents with Developmental Disabilities on Disability-Related Factors Influencing Their Infant Feeding Decisions: A Mixed Methods Study

**DOI:** 10.3390/nu18111674

**Published:** 2026-05-23

**Authors:** Stacy V. Lu, Susan M. Gross, Allison L. West

**Affiliations:** 1 College of Health Sciences, University of Memphis, 161 Elma Roane Fieldhouse, Memphis, TN 38152, USA; 2 Department of Population, Family and Reproductive Health, Johns Hopkins Bloomberg School of Public Health, 615 N Wolfe St., Baltimore, MD 21205, USA

**Keywords:** infant feeding, breastfeeding, parents with developmental disabilities, disabled parenting, mixed methods, parent perspectives

## Abstract

**Background/Objectives**: The practices that parents use to feed their infants have important implications for life course health and well-being. However, little is known about the infant feeding experiences and decisions of parents with developmental disabilities. This study used a mixed methods design to gain an in-depth understanding of the infant feeding experiences and decisions of parents with developmental disabilities in the United States. **Methods**: Between July 2024 and June 2025, 18 parents with developmental disabilities completed a one-time quantitative survey, seven of whom also completed three individual qualitative interviews. Analytical procedures included descriptive statistics of quantitative survey data and thematic analysis of qualitative interviews, followed by integration of the two forms of data. All interview participants completed member checking of preliminary themes. **Results**: Parents with developmental disabilities described varied experiences with breastfeeding, formula feeding, and introducing solid foods to their infants at around six months. Four disability-related factors influenced parents’ decisions across different infant feeding practices: (1) sensitivity to sensory stimuli; (2) demands on executive function; (3) “rigid thinking” about breastfeeding; and (4) medication use. **Conclusions**: Findings suggest parents with developmental disabilities may benefit from direct and customized support with infant feeding. Changes to improve access to disability-affirming care are warranted.

## 1. Introduction

Breastfeeding is a natural and critically important infant feeding practice proven to offer several short- and long-term benefits to infant and parent health [1,2,3,4]. The World Health Organization, therefore, recommends exclusive breastfeeding during an infant’s first six months, followed by the introduction of complementary solid foods at six months alongside continued breastfeeding through the first two years or longer [5]. If breastfeeding is not possible, the use of commercial milk formula is the only acceptable alternative as the sole or supplementary feeding for infants through the first year [6,7]. Introducing solid foods to infants before four months is considered too early and precludes the recommended six months of exclusive breastfeeding.

Despite strong evidence of the health benefits and global recommendations, in the United States (US), under 30% of infants are exclusively breastfed at six months and less than half are breastfed at all at one year [8]. Suboptimal breastfeeding prevalence in the US is due, in part, to persistent social and structural barriers that are unsupportive of breastfeeding [9,10,11,12]. Substantial evidence shows systemically marginalized populations face additional or exacerbated barriers, contributing to disparities in infant feeding outcomes as well as parent and infant health outcomes [13,14,15,16,17]. One such population is parents with developmental disabilities, but their infant feeding experiences and support needs have received minimal attention in public health research and guidance to date [18,19].

Data on the infant feeding practices of parents with developmental disabilities is inherently limited by the overall lack of data on parents with developmental disabilities. Current estimates suggest that at least 6.7% of the US adult parenting population has a disability [20], but the national prevalence of parents with developmental disabilities is unknown due to measurement limitations [21,22,23].

Prior research on infant feeding among US parents with developmental disabilities has specifically focused on breastfeeding. One study found women with intellectual and developmental disabilities have a lower prevalence of breastfeeding at hospital discharge compared to women with diabetes—a disability but not necessarily a developmental disability—and women with neither diabetes nor intellectual and developmental disabilities (49.4% vs. 74.2% vs. 77.4%) [24]. Another study found parents with disabilities—including parents with developmental disabilities—have lower prevalences of any breastfeeding since birth (83.1% vs. 87.0%) and at one month (68.7% vs. 76.7%), two months (56.1% vs. 67.0%), and three months after birth (48.3% vs. 60.0%) compared to parents without disabilities [25]. In qualitative studies, parents with developmental disabilities have reported barriers to breastfeeding, such as a lack of information and support from health care providers about disability-related needs and experiences with breastfeeding [26,27,28,29,30,31].

Extant research is scarce and does not fully explain infant feeding disparities among parents with developmental disabilities. Prior research has identified several factors across socioecological levels that influence the infant feeding decisions of many parents in general, including cost, workplace and hospital policies, social and professional support, and individual intentions (i.e., intention to breastfeed) [2,9,12,16,32,33,34]. However, little is known about the factors that uniquely shape the infant feeding decisions of parents with developmental disabilities and their experiences with different infant feeding practices overall. There is a need for further research to better understand the breastfeeding experiences of parents with developmental disabilities as well as their experiences with other infant feeding practices, such as formula feeding and the introduction of solid foods. The ways in which parents with developmental disabilities experience and make decisions about feeding formula and solid foods have important implications for breastfeeding exclusivity and duration but have not been investigated deeply or at all. Research is needed to capture the different practices that parents with developmental disabilities use to feed their infants and gain a holistic understanding of their infant feeding experiences and decisions. Such research is a necessary starting point to address life course health disparities by identifying opportunities for targeted interventions and changes to program and practice guidelines to better support parents with developmental disabilities in making and implementing decisions about how to feed their infants. The perspectives of parents with developmental disabilities are essential to ensure such changes are affirming and effective; however, research centering their perspectives is scarce.

### Current Study

This article presents findings from a broader study that centered on the perspectives of US parents with developmental disabilities to understand their infant feeding experiences, their disability identity in the context of infant feeding, and the extent to which known factors are adequate for explaining their infant feeding decisions. In this article, we focus on describing the infant feeding practices and disability-related factors influencing infant feeding decisions among US parents with developmental disabilities.

## 2. Materials and Methods

### 2.1. Study Design

**Figure 1:**
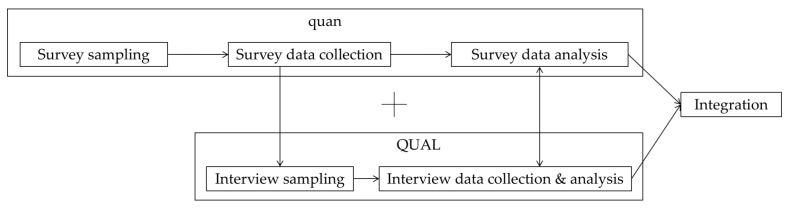
Diagram of study design.

The broader study used a qualitatively driven concurrent mixed methods design consisting of two research strands (quan + QUAL) (Figure 1). Data collection methods included a one-time survey in the quantitative strand and three individual interviews in the qualitative strand. The two strands occurred simultaneously, such that the collection and analysis of quantitative and qualitative data influenced each other, and the qualitative sample was recruited from the larger quantitative sample. A constructivist paradigm guided the overall design, meaning the study emphasized dialogic and participatory approaches to explore participants’ perspectives [35,36]; thus, the study prioritized the qualitative strand. The study was approved by the Institutional Review Board of the Johns Hopkins Bloomberg School of Public Health.

### 2.2. Eligibility Criteria

Study eligibility criteria included age 18 years or older, at least one live-born infant who was age 12 months or younger at the time of the survey, current residence in the US or US territory, and self-reported developmental disability from a provided list (Table 1). Additional criteria included the ability to read or speak in English and being the birth or biological parent, adoptive parent, or stepparent of the infant. We included a broad and diverse range of developmental disabilities without requiring clinical diagnoses to be as inclusive as possible and maximize recruitment of a population that has been historically excluded from research.

### 2.3. Procedures

#### 2.3.1. Quantitative Survey Procedures

We used convenience sampling to first recruit survey participants by distributing a digital recruitment flyer primarily through social media and online networks of organizations engaged in disability-related work across the US. We also used snowball sampling by asking interview participants to share the recruitment flyer with others who they knew may be interested and eligible for the study.

Interested individuals completed an anonymous online eligibility screener, after which eligible individuals automatically proceeded to the online consent form, and consented individuals automatically proceeded to the online survey. We reviewed responses to confirm eligibility and remove suspected bots and scammers. Participants could opt into a raffle for one of two $25 gift cards after completing the survey. All online tools were administered via REDCap hosted at Johns Hopkins University [37,38].

A total of 18 participants completed the survey. The survey opened in July 2024 and closed in June 2025. However, most participants completed the survey between October 2024 and February 2025. Survey completion time ranged from four to 28 min.

#### 2.3.2. Qualitative Interview Procedures

At the end of the survey, participants could express their interest in participating in an initial interview. The first author contacted all who expressed interest to schedule a 15 to 20 min pre-interview call to confirm interest and eligibility and obtain ongoing consent. Ongoing consent for the second and third interviews employed similar procedures. Interviews were scheduled at the participant’s convenience, offered over the phone or on Zoom, audio-recorded, and professionally transcribed. Participants received a $30 gift card after completing each interview. The first author conducted all interviews and, with assistance from a trained student researcher, checked each interview transcript against the recording for accuracy and edited for anonymity.

Seven of the 18 survey participants agreed to participate in the interviews; all interview participants completed the entire series of three interviews. Interview participants completed their initial interview within one month of survey completion, with the first initial interview conducted in August 2024 and subsequent initial interviews conducted in January and February 2025. All interview participants completed their second interview in May 2025 and their third interview in June 2025. Initial interviews lasted from 40 to 60 min, second interviews lasted from 33 to 60 min, and third interviews lasted from 23 to 44 min.

### 2.4. Measures

#### 2.4.1. Quantitative Survey Measures

We developed an online survey with up to 65 questions asking about participants’ demographic characteristics, infant feeding practices, and literature-based factors that may have influenced their infant feeding decisions, including social determinants of health. Below, we describe survey measures specifically used in the analyses presented in this article.

Demographic Characteristics: Items in the eligibility screener assessed the participant’s type of developmental disability, their age (continuously in years) and their infant’s age (categorically in months) at the time of the survey, relationship to the infant, the number of infants ≤12 months they had (dichotomized into one or more than one), and plurality of the birth from which the infant was born (dichotomized into singleton or multiple). Additional survey items assessed the participant’s race/ethnicity, gender, marital status, educational attainment, employment status, length of residence in the US (continuously in years), and whether the participant was the person who gave birth to the infant.

Social Determinants of Health: The survey assessed nine social determinants of health: food security risk, receipt of federal nutrition assistance, energy security, housing needs, transportation needs, childcare needs, financial strain, experiences of discrimination, and experiences of violence. Housing and childcare needs were assessed at the time of the survey, and all others in the past 12 months.

A two-item screener assessed food security risk [39]: “In the past 12 months, did you worry that food at home would run out before your family got money to buy more?” and “In the past 12 months, did the food that your family bought run out and you didn’t have money to get more?” (0 = Never; 1 = Sometimes; 2 = Often). Participants were categorized as at risk of experiencing food insecurity if they answered “sometimes” or “often” to at least one item.

A single item from the American Community Survey assessed receipt of benefits from the Supplemental Nutrition Assistance Program (SNAP) [40]: “In the past 12 months, did you or anyone in your household receive benefits from the Food Stamp Program or Supplemental Nutrition Assistance Program (SNAP)?” (0 = No; 1 = Yes). Based on this item, we developed a separate item to assess receipt of benefits from the Special Supplemental Nutrition Program for Women, Infants, and Children (WIC): “In the past 12 months, did you or anyone in your household receive benefits from Special Supplemental Nutrition Program for Women, Infants, and Children (WIC)?” (0 = No; 1 = Yes). Participants were categorized as receiving federal nutrition assistance if they answered affirmatively to at least one item.

We assessed energy security and financial strain using items from the Social Needs Screening Tool by the American Academy of Family Physicians [41]. The energy security item asked: “In the past 12 months, did the electric, gas, oil, or water company threaten to shut off services in your home?” (0 = No; 1 = Yes; 2 = It was already shut off). Participants were categorized as energy insecure if they answered “yes” or “it was already shut off.” To assess financial strain, we modified an item to avoid Likert-scale response options and improve understandability [41], following recommendations by Shogren et al. [42]: “In the past 12 months, did you always have enough money to pay your bills?” (0 = No; 1 = Yes).

We also modified and simplified items from the phase nine questionnaire of the Pregnancy Risk Assessment Monitoring System to assess transportation needs and experiences of discrimination [43]. The transportation item asked: “In the past 12 months, did lack of transportation keep you from medical appointments, meetings, work, or from getting things needed for daily living?” (0 = No; 1 = Yes). The discrimination item asked: “In the past 12 months, has anyone ever judged you unfairly or discriminated against you?” (0 = No; 1 = Yes, because of my developmental disability; 2 = Yes, because of another disability or health condition; 3 = Yes, because of my use of medications for my developmental disability; 4 = Yes, because of my use of medications for another disability or health condition; 5 = Yes, because of my race, ethnicity, and/or skin color; 6 = Yes, because of my gender or gender identity; 7 = Yes, because of my sexuality or sexual orientation; 997 = Yes, because of another reason; 998 = I do not know or I am unsure). Based on this item, we developed a separate item to assess experiences of violence using the same response options: “In the past 12 months, has anyone ever harmed, insulted, or threatened you?” Participants were categorized as experiencing discrimination or violence if they selected at least one affirmative answer to each item.

To assess childcare needs, we modified an item from the Children’s HealthWatch Survey for the purpose of our study [44]: “Do problems getting childcare ever make it difficult for you to do errands or go to appointments, meetings, school, or work?” (0 = No; 1 = Yes). Finally, we developed a single item to assess housing needs: “Do you currently have housing?” (0 = No; 1 = Yes).

Infant Feeding Practices: Guided by global infant and young child feeding indicators [4], we developed survey items to assess participants’ infant feeding practices since birth and at the time of survey completion. Practices included chest/breastfeeding (defined as any type of human milk), formula feeding (defined as any type of commercial infant formula), feeding other liquids besides human milk or formula (e.g., cow’s milk, juice), and feeding soft, semi-solid, or solid foods (e.g., baby cereal, eggs).

A single item assessed chest/breastfeeding since birth: “From the time your baby was born until now, what are all the ways your baby has been fed breast milk or chest milk?” (0 = Never fed breast milk or chest milk; 1 = From the breast or chest; 2 = From a bottle, syringe, spoon, or cup; 997 = In another way not listed here; 998 = I do not know or I am unsure). Data were recoded as a dichotomous variable (0 = None; 1 = Any).

An additional item assessed the source of human milk among participants who indicated at least some chest/breastfeeding since birth: “From the time your baby was born until now, what are all the people or places where you have gotten breast milk or chest milk for your baby?” (1 = I breastfed or chest-fed; 2 = I pumped milk; 3 = My partner/co-parent breastfed or chest-fed; 4 = My partner/co-parent pumped milk; 5 = I got breast milk or chest milk from a donor milk bank; 6 = I got breast milk or chest milk from a friend, relative, or in-law; 997 = I got breast milk or chest milk from another person or place not listed here; 998 = I do not know or I am unsure). Responses were recoded for ease of interpretation (1 = Nursed only; 2 = Pumped only; 3 = Nursed and pumped; 4 = Got donor milk).

A single item assessed formula feeding since birth: “From the time your baby was born until now, what are all the types of infant formula that have been fed to your baby?” (0 = Never fed infant formula; 1 = Liquid formula; 2 = Powdered formula; 997 = Another type of formula not listed here; 998 = I do not know or I am unsure). Data were recoded as a dichotomous variable (0 = None; 1 = Any).

Single items assessed chest/breastfeeding, formula feeding, feeding other liquids, and feeding solid foods at the time of the survey: “Is your baby being fed breast milk or chest milk/infant formula/other liquids/solid foods now?” (0 = No; 1 = Yes; 998 = I do not know or I am unsure). Data were recoded as a single categorical variable for ease of interpretation (1 = Any chest/breastfeeding; 2 = Any formula feeding; 3 = Exclusively chest/breastfeeding; 4 = Exclusively chest/breastfeeding; 5 = Exclusively formula feeding; 6 = Combination of chest/breastfeeding and formula feeding; 7 = Any other liquids introduced; 8 = Any solid foods introduced).

A single item assessed the addition of solid foods to a feeding bottle among participants who indicated introducing at least some solid foods at the time of the survey: “Do you ever add solid foods to the bottle?” (0 = No; 1 = Yes; 996 = This does not apply because my baby has never been fed from a bottle; 998 = I do not know or I am unsure).

#### 2.4.2. Qualitative Interview Measures

For the initial interview, we designed a semi-structured interview guide containing 11 open-ended questions to build on the participants’ survey responses and elaborate on their infant feeding experiences and the factors influencing their decisions (Table A1). For the second interview, we designed a loosely structured interview guide containing five open-ended questions to follow up on the participant’s previous responses (Table A2). We referenced questions in interview guides used in prior qualitative studies on similar topics and populations to guide the development of questions in our interview guides [27,31,45,46,47,48]. Additionally, prior to launching the study, we tested the initial interview guide with two parents with young children: one mother without disabilities and one father with developmental disabilities. Minor revisions to the phrasing of questions were made based on their feedback.

The purpose of the third interview was for member checking, a data verification strategy in which participants provide their feedback on the accuracy of the findings [49]. Guided by the member checking approach described by McKim [50], we designed a loosely structured interview guide containing five open-ended questions to solicit participants’ feedback on preliminary themes from the interviews (Table A3).

### 2.5. Data Analysis

Descriptive statistics of survey data were calculated using R Statistical Software v. 4.5.0 (Vienna, Austria) via the dplyr and psych packages [51,52,53]. Interview data were analyzed using MAXQDA 2024 (Berlin, Germany) [54]. The first author employed a combination of inductive and deductive strategies to develop a codebook with codes derived directly from the interview data (inductive) and organized them into categories and themes that corresponded with the survey data and study aims (deductive). Tables were created to jointly display survey and interview data side-by-side, aid in interpreting the two forms of data together, and explore infant feeding decision-making factors not captured in the survey.

For the member checking interviews, the first author reviewed interview participants’ feedback on the preliminary themes individually and across participants. Based on feedback from two interview participants, minor revisions were made to the description of themes to better capture the full range of their thoughts and experiences. Otherwise, participants felt that their perspectives were well-captured overall.

Throughout the study, the first author engaged in peer debriefing and recorded memos to support ongoing analysis and engage in reflexivity, a practice in which the researcher critically reflects on the data [55,56].

## 3. Results

### 3.1. Participant Characteristics

This section is primarily informed by the quantitative survey and describes the demographic characteristics (Table 2) and social determinants of health (Table 3) of parents with developmental disabilities.

All 18 participants gave birth to their infants. At the time of the survey, participants’ ages ranged from 21 to 38 years, half (*n* = 9) had infants under six months old, and slightly over half were employed (*n* = 10; 55.6%). Two participants gave birth to multiple infants (i.e., twins, triplets) (11.1%); all others gave birth to singleton infants. Most participants lived in the US for their entire lives (*n* = 16; 88.9%), identified as white or European American (*n* = 16; 88.9%), identified as women (*n* = 15; 83.3%), were married (*n* = 14; 77.8%), and completed at least some college or training school (*n* = 17; 94.4%). Participants self-reported having one or more of the following developmental disabilities: ADHD/ADD (*n* = 12), autism/ASD (*n* = 9), language disorder (*n* = 1), learning disorder (*n* = 2), and another developmental disability not listed (*n* = 4). When asked to describe their other developmental disability, participants named AUTS2 syndrome (*n* = 1), auditory processing disorder (*n* = 1), sensory processing disorder (*n* = 1), and thrombocytopenia-absent radius (TAR) syndrome (*n* = 1). Based on information from the interviews, interview participants lived in seven different states across all four regions of the US, and all referred to themselves as mothers, two were first-time mothers, and five had clinical diagnoses for their developmental disabilities.

At the time of the survey, no participants reported housing needs, and seven (38.9%) reported childcare needs. In the past 12 months since the survey, no participants reported transportation needs, six (33.3%) experienced a risk of food insecurity, one (5.6%) received federal nutrition assistance from WIC, two (11.1%) experienced energy insecurity, four experienced financial strain (22.2%), 11 (61.1%) experienced at least one instance where another person unfairly judged or discriminated against them, and three (16.7%) experienced at least one instance where another person harmed, insulted, or threatened them.

### 3.2. Infant Feeding Practices of Parents with Developmental Disabilities

This section is based on the quantitative survey data and describes the infant feeding practices of parents with developmental disabilities (Table 4). All 18 participants chest/breastfed their infants at least some since birth, and 14 (77.8%) fed at least some formula since birth. All seven participants with infants older than six months introduced at least some other liquids or solid foods to their infants at the time of the survey; five (71.4%) participants also chest/breastfed at least some.

### 3.3. Disability-Related Factors That Influenced the Infant Feeding Decisions of Parents with Developmental Disabilities

This section is based on the qualitative interview data and describes disability-related factors that influenced how parents with developmental disabilities made decisions about the infant feeding practices identified from the quantitative survey data. Four disability-related factors were identified:

1. Sensitivity to sensory stimuli;
2. Demands on executive function;
3. “Rigid thinking” about breastfeeding;
4. Medication use.

#### 3.3.1. Sensitivity to Sensory Stimuli

Many participants described having a sensitivity to sensory stimuli, such as noise and touch, which they attributed to heightened difficulties with pain or discomfort while breastfeeding and unpleasant textures and messiness while feeding solid foods. An autistic and ADHD mother shared:

I would, a lot of times, just breastfeed [my baby] in the beginning, but I would sit there and I would cry while he was feeding. […] It was so painful, and it made my entire body feel activated and stiff and touched out. […] It was like a snowball effect because […] I was running out of internal resources to feel the other things that I might have been able to tolerate better had I not been going through that with the breastfeeding. (Infant age at initial interview: 5 months)

A mother with ADHD, dyslexia, and auditory processing disorder added:

One of my biggest problems is I have really struggled with getting messy and you know, [laughs] feeding a baby, you get messy. He gets messy. Everybody gets messy. I feel like that’s kind of something every time I feed him, I have to work up to it because it’s not a very pleasant sensation. (Infant age at initial interview: 7 months)

Participants had mixed experiences with the relative sensory load of different breastfeeding practices. For example, some participants found nursing less overstimulating than pumping while others found pumping less overstimulating than nursing. A mother with autism, ADHD, language disorder, and AUTS2 syndrome explained:

Well, sensory issues for me made it very scary to breastfeed just because I didn’t know if I’d be able to sit still long enough or tolerate it long enough. And so that made it not feel like a real option, but I did breastfeed in a way. I just didn’t breastfeed-breastfeed. I pumped. So, it’s not like [my baby] didn’t get breastfed. It’s just that they didn’t get breastfed on the breast. (Infant age at initial interview: 3 months)

Some participants viewed formula feeding as a recourse from breastfeeding because it was less overstimulating than nursing and pumping. An autistic mother shared:

I feel like maybe with autism, the sensory thing of sometimes I just don’t feel like always getting my shirt soaked in milk and spit it up and having that sensation, and all the touch overstimulation and everything. So, it’s nice to have the option to do formula for whatever reason. (Infant age at initial interview: 3 months)

Regardless of feeding practice, participants consistently preferred and often chose the most sensorily tolerable option. Additionally, several participants reported using strategies to make various infant feeding practices less overstimulating. Examples included wearing looser, larger clothing or noise-canceling headphones while breastfeeding and feeding big chunks of food instead of just purees when introducing solid foods.

#### 3.3.2. Demands on Executive Function

Several participants described difficulties with executive function related to their developmental disabilities, such as trouble concentrating and completing tasks. Participants opted for feeding practices that they perceived to be simple, convenient, and predictable or routine. For example, although participants had mixed experiences with the routineness of breastfeeding, they agreed breastfeeding offered advantages of reduced executive functioning demands due to its relative simplicity and convenience compared to other feeding practices. A mother with ADHD, dyslexia, and auditory processing disorder explained:

I think breastfeeding is especially convenient for people with ADHD because I don’t have to have the patience to wait for the bottle to warm up. [laughs] … If I’m going out, I don’t have to make sure I’ve got the formula and the bottles and the way to heat it up and all that so it definitely takes some of that mental load away, so less planning involved. (Infant age at initial interview: 7 months)

However, when it came time to introduce solid foods, some participants struggled with the transition to a different routine, especially when breastfeeding was mutually preferred by both the participant and their infant. An autistic and ADHD mother explained:

For me, introducing new routines is really difficult. I think that’s just the ADHD part of me. […] It actually just became easier to breastfeed. It was like this is now less stress to just offer [my baby] the breast and not even try to feed him solids today. That’s a slippery slope. Now I have a nine-and-a-half-month-old that barely eats solids. (Infant age at initial interview: 5 months)

#### 3.3.3. “Rigid Thinking” About Breastfeeding

Some participants described having a tendency toward rigid thinking or fixating on the idea that “breast is best.” This motivated participants to educate themselves about breastfeeding and establish breastfeeding goals, but it also increased the stress associated with making decisions. An autistic mother elaborated:

I think, with autism or other developmental disabilities, there can be a tendency towards rigid thinking and being really self-critical. That makes the breastfeeding or not breastfeeding journey really difficult of knowing this breastfeeding for a year or two years, however long, is the right thing to do. It was added stress to not be able to sustain it or choose not to. Knowing that’s not the most ideal thing is pretty stressful. (Infant age at initial interview: 3 months)

#### 3.3.4. Medication Use

For many participants, breastfeeding decisions were intertwined with decisions about using medications for their developmental disabilities or other disabilities. Most often, participants decided to stop taking medications so that they could breastfeed without worrying about any potential risks to their infant. An ADHD mother shared:

[Before my pregnancy] I did [take ADHD medications], but most of them are not pregnancy-safe. Some of them are breastfeeding-safe, but I try not to introduce any new medications if I don’t absolutely need to, especially ones that are going to put my [baby] at any sort of risk. So, I am holding off for now, but I am interested to kind of see what my options are once I am done breastfeeding and hopefully see some improvement in my mental health. (Infant age at initial interview: 2 months)

Offering a different perspective, one mother with autism, ADHD, language disorder, and AUTS2 syndrome explained she never breastfed her older children because she assumed she could not do so while taking medications:

I take multiple different psychiatric medications. Because of that, I never thought [breastfeeding] was a possibility. […] The primary reason I chose not to breastfeed or even really look into it was because with [my oldest child], I was finally stable on the medications I was on and I didn’t want them, if I had a psychotic breakdown or something like that, to have to worry about breastfeeding. […] I had kind of just decided that I wouldn’t breastfeed because I thought it would be easier on everybody. (Infant age at initial interview: 3 months)

## 4. Discussion

This mixed methods study expands upon a limited but growing body of evidence focused on improving infant feeding services and supports for parents with developmental disabilities. The study centered on the perspectives of parents with developmental disabilities through a quantitative survey and three qualitative interviews, the integration of which enabled the exploration of infant feeding decision-making factors that would not have been captured using either research method alone. Findings show ways in which parents’ developmental disabilities may shape their experiences and decisions with infant feeding.

Breastfeeding difficulties, such as pain or discomfort and pragmatic concerns related to the inability to maintain a predictable schedule are commonly cited reasons for breastfeeding cessation among many parents, not just parents with developmental disabilities [57]. However, as parents in this study explained, breastfeeding difficulties may be exacerbated for parents with developmental disabilities, in part due to sensitivity to sensory stimuli and executive functioning demands. Additionally, the inability to breastfeed exclusively or at all may be particularly distressing for parents with developmental disabilities who intensely internalize expectations of best practices. Parents’ breastfeeding decisions, or cessation thereof, are also tied to their decisions about whether to continue or discontinue medications for their developmental disabilities or other disabilities. These findings suggest parents with developmental disabilities may benefit from infant feeding information and counseling that considers how their developmental disabilities shape their infant feeding experiences and decisions.

Findings from this study lend additional support for improving access to disability-affirming resources and counseling with infant feeding. Regardless of feeding practice, all parents must be educated and supported to safely feed their infants. The National Center for Disability and Pregnancy Research offers research-informed resources—including one focused on breastfeeding—created by and for disabled parents [58]. Recommendations emphasize keeping disabled parents comfortable, ensuring resources are accessible to parents, and facilitating open conversations with health care providers about parents’ disability-related needs. Parents with developmental disabilities, in particular, may need support with finding strategies to minimize demands on sensory load and executive function while feeding their infants. However, resources that consider the specific needs of parents with developmental disabilities are currently lacking. Additionally, many providers who deliver direct services to pregnant and postpartum parents with developmental disabilities do not feel adequately prepared to provide care to this population, and available teaching materials may be inaccessible [59,60]. Changes to increase and systematize formal opportunities for providers to gain the necessary tools, skills, and knowledge to better support parents with developmental disabilities with infant feeding are warranted. Additional research is needed to inform such changes, and future research must directly engage parents with developmental disabilities.

In this study, parents with developmental disabilities expressed concerns about taking medications while breastfeeding. They described a tradeoff between their own health and their infant’s health. Providers must be adequately trained to provide all parents with accurate information about the risks and benefits of medication use while breastfeeding to make informed decisions. Based on our findings, it may be helpful for providers to discuss parents’ decisions about medications and breastfeeding as a joint process, rather than separately. It is possible that some parents in this study may have avoided breastfeeding or discontinued medications when it was not contraindicated. These findings also elicit concerns about the accuracy of information presented to parents with developmental disabilities on medication safety while breastfeeding.

### Strengths and Limitations

The strengths and limitations of this study are deeply intertwined, and findings should be interpreted in consideration of these strengths and limitations. By collecting and validating data directly with parents with developmental disabilities, the study addressed an urgent gap in current research and practice while elevating the perspectives of a population that has been historically neglected and excluded from research. The use of mixed methods enabled integration of quantitative survey data and qualitative interview data. Prolonged engagement and member checking with interview participants enhanced the trustworthiness of the study findings.

A key strength of the study is the successful recruitment of a highly stigmatized and hard-to-reach population. The study used an intentionally broad sampling strategy based on self-reported developmental disability, which allowed for a diversity of participants with different types of developmental disabilities, including those with and without clinical diagnoses. This enabled participation by parents who may have been excluded or deterred from participating based on stricter criteria. However, important differences may exist between parents with and without clinical diagnoses and may have implications for their infant feeding experiences and decisions, such as access to prescription medications while chest/breastfeeding. Furthermore, despite efforts to recruit a demographically diverse sample, the study recruited a small convenience sample in which the majority were highly educated, married, cis-gender white women who lived in the US for all or most of their lives. Parents with developmental disabilities who also belong to other marginalized communities may be harder to reach. Findings may not reflect the perspectives of parents with developmental disabilities who were disinclined to participate in the study for whatever reason. Additionally, the study did not collect data on participants’ partners or co-parents, some of whom may also have developmental disabilities, and should be included in future research. Finally, parents with developmental disabilities were not involved during the design and implementation of the study, which is a recommended practice [61].

## 5. Conclusions

Parents with developmental disabilities may benefit from direct and customized support with infant feeding that considers how their disabilities may shape their experiences and decisions. There is a need to improve access to disability-affirming care, such as through formal opportunities for provider education and training on the infant feeding support needs of parents with developmental disabilities. Future efforts must center the perspectives of parents with developmental disabilities to ensure research, programs, and practices are designed and implemented in ways that are affirming and effective.

## Figures and Tables

**Table 1:** Study-provided list of developmental disabilities.

Attention-deficit/hyperactivity disorder (ADHD) or attention-deficit disorder (ADD)
Autism or autism spectrum disorder (ASD)
Cerebral palsy
Down syndrome
Fetal alcohol spectrum disorders
Fragile X syndrome
Intellectual disability
Language disorder (e.g., aphasia)
Learning disorder (e.g., dyslexia)
Tourette syndrome
A developmental disability not represented in this list ^1^

^1^ If selected, potential participants described their developmental disability in an open text field.

**Table 2:** Demographic characteristics of parents with developmental disabilities: overall (*n* = 18) and by interview participation (*n* = 7).

Characteristic	Overall (*n* = 18)		Interview Participants (*n* = 7)	
	*n* or Mean	% or SD	*n* or Mean	% or SD
Age (years)	30.5	4.1	32.1	4.3
Length of U.S. residence (years)	28.8	8.1	32.1	4.3
Developmental disability				
ADHD/ADD	12	66.7	4	57.1
Autism/ASD	9	50.0	5	71.4
Language disorder	1	5.6	1	14.3
Learning disorder	2	11.1	2	28.6
Another developmental disability not listed	4	22.2	3	42.9
Race/ethnicity				
White or European American	16	88.9	7	100.0
Hispanic, Latine/x, or Spanish	3	16.7	1	14.3
American Indian or Native American	1	5.6	1	14.3
Black or African American	1	5.6	0	0
Gender				
Woman	15	83.3	6	85.7
Cisgender	1	5.6	1	14.3
Nonbinary	2	11.1	0	0
Marital status				
Married	14	77.8	7	100.0
Living with a partner but not married	3	16.7	0	0
Single, widowed	1	5.6	0	0
Education				
Less than HS	1	5.6	0	0
Some college or training school	4	22.2	2	28.6
College/technical school degree	10	55.6	4	57.1
Graduate/doctoral degree	3	16.7	1	14.3
Employed	10	55.6	3	42.9
More than 1 infant ≤12 months	3	16.7	0	0
Youngest infant came from singleton birth	16	88.9	7	100.0
Participant gave birth to infant(s)	18	100.0	7	100.0
Age of infant(s)				
≤6 months	11	61.1	5	71.4
<1 month	1	5.6	0	0
1–3 months	7	38.9	4	57.1
4–6 months	3	16.7	1	14.3
>6 months	7	38.9	2	28.6
7–9 months	4	22.2	1	14.3
10–12 months	3	16.7	1	14.3

**Table 3:** Social determinants of health among parents with developmental disabilities: overall (*n* = 18) and by interview participation (*n* = 7).

Determinant	Overall (*n* = 18)		Interview Participants (*n* = 7)	
	*n*	%	*n*	%
Participant experienced risk of food insecurity	6	33.3	2	28.6
Receipt of federal nutrition assistance				
Any	1	5.6	0	0
None	15	83.3	7	100.0
Missing	2	11.1	0	0
Participant experienced energy insecurity	2	11.1	0	0
Participant had housing needs	0	0	0	0
Participant had transportation needs	0	0	0	0
Participant had childcare needs	7	38.9	2	28.6
Financial strain				
Yes	4	22.2	2	28.6
No	13	72.2	5	71.4
Experiences of discrimination				
Any	11	61.1	5	71.4
Developmental disability	7	38.9	3	42.9
Medication use for developmental disability	2	11.1	1	14.3
Another disability	1	5.6	1	14.3
Medication use for another disability	1	5.6	0	0
Race/ethnicity/skin color	2	11.1	1	14.3
Gender/gender identity	2	11.1	2	28.6
Another reason	1	5.6	1	14.3
None	6	33.3	2	28.6
Don’t know/unsure	1	5.6	0	0
Experiences of violence				
Any	3	16.7	1	14.3
Developmental disability	1	5.6	0	0
Gender/gender identity	1	5.6	1	14.3
Another reason	1	5.6	0	0
None	13	72.2	5	71.4
Don’t know/unsure	1	5.6	0	0
Missing	1	5.6	1	14.3

**Table 4:** Infant feeding practices used by parents with developmental disabilities (*n* = 18), categorized by infant age ≤6 months (*n* = 11) vs. >6 months (*n* = 7).

Practice	Overall (*n* = 18)		≤6 Months (*n* = 11)						>6 Months (*n* = 7)			
			<1 Month		1–3 Months		4–6 Months		7–9 Months		10–12 Months	
	*n*	%	*n*	%	*n*	%	*n*	%	*n*	%	*n*	%
Since birth												
Any chest/breastfeeding	18	100.0	1	9.1	7	63.6	3	27.3	4	57.1	3	42.9
Nursed only	2	11.1	0	0	0	0	0	0	2	28.6	0	0
Pumped only	3	16.7	0	0	2	18.2	0	0	0	0	1	14.3
Nursed and pumped	13	72.2	1	9.1	5	45.4	3	27.3	2	28.6	2	28.6
Got donor milk	2	11.1	0	0	2	18.2	0	0	0	0	0	0
Any formula feeding ^1^	14	77.8	0	0	6	54.4	3	27.3	3	42.9	2	28.6
At time of survey												
Any chest/breastfeeding	14	77.8	1	9.1	6	54.4	2	18.2	3	42.9	2	28.6
Any formula feeding	9	50.0	0	0	5	45.4	2	18.2	1	14.3	1	14.3
Exclusively chest/breastfeeding	4	22.2	1	9.1	2	18.2	1	9.1	0	0	0	0
Exclusively formula feeding	2	11.1	0	0	1	9.1	1	9.1	0	0	0	0
Combination of chest/breastfeeding and formula feeding	5	27.8	0	0	4	36.4	1	9.1	0	0	0	0
Any other liquids introduced	6	33.3	0	0	0	0	1	9.1	1	14.3	0	0
Any solid foods introduced	8	44.4	0	0	0	0	1	9.1	4	57.1	3	42.9
Added to bottle	1	5.6	0	0	0	0	0	0	1	14.3	0	0

^1^ One participant did not respond to the survey item assessing formula feeding since birth.

## Data Availability

Deidentified data may be available from the corresponding author upon request due to privacy reasons.

## Abbreviations

The following abbreviations are used in this manuscript:

US	United States
ADHD	Attention-deficit/hyperactivity disorder
ADD	Attention-deficit disorder
ASD	Autism spectrum disorder
SNAP	Supplemental Nutrition Assistance Program
TAR	Thrombocytopenia-absent radius
WIC	Special Supplemental Nutrition Program for Women, Infants, and Children

## Appendix A. Interview Guides

**Table A1:** Initial interview guide.

In this interview, I want to hear about your experiences being a parent with developmental disabilities and how you feed your baby. First, I want to know more about you. How are you today? 1. In your survey, you mentioned [insert disability]. Tell me more. 2. What is it like being a parent? 3. What does being a parent with [insert disability] mean to you? Thank you. Now I want to ask about your experiences with feeding your baby. 4. Tell me about your baby. 5. In your survey, you mentioned [insert infant feeding practices]. Tell me about what it was/is like for you. 6. How did you come to the decision to feed your baby this way? If needed: Tell me like it’s a story about your journey with feeding your baby. 7. What are the big reasons why you fed/feed your baby this way? 8. Are there other ways you feed your baby that we haven’t talked about? For example, mixing breast/chest milk with formula? 9. What is it like for you to be a parent with [insert disability] while feeding your baby? If needed: How has being a parent with [insert disability [affected how you feed your baby? I just have a few more questions before we wrap up. 10. What do you think are some ways that could help other parents with developmental disabilities with feeding their babies? If needed: Is there any advice you would give to other parents with developmental disabilities to help them with feeding their babies? Is there any advice you would give to medical or lactation professionals to better support parents with developmental disabilities with feeding their babies? 11. Is there anything else you’d like to tell me about yourself or how you decided to feed your baby?

**Table A2:** Second interview guide.

In this interview, I want to hear more about your experiences being a parent with developmental disabilities and how you feed your baby. We are going to follow up on what we talked about in your first interview. First, how are you today? 1. Tell me about how you and your baby have been since we last talked. 2. In your first interview, you mentioned [insert disability experiences]. Tell me more. Thank you. Now I want to ask about your experiences with feeding your baby. 3. In your first interview, you mentioned [insert infant feeding experiences]. Tell me more. Thank you. Now I want to ask about your experiences with feeding your baby. I just have a few more questions before we wrap up. 4. In your first interview, you mentioned [insert recommendations]. Tell me more. 5. Is there anything else you’d like to tell me about yourself or how you decided to feed your baby?

**Table A3:** Third (member checking) interview guide.

In this interview, I’m going to share what I learned from you and the other parents in the study and ask you some questions. I want to hear your thoughts on what I found. First, how are you today? 1. One thing I learned is that [insert summary of findings on infant feeding practices]. What are your thoughts? 2. I also learned that [insert summary of findings on disability identity]. What are your thoughts? 3. Finally, I learned about [insert summary of findings on factors influencing infant feeding decisions]. What are your thoughts? Thank you for talking through the findings with me. I just have a few more questions before we wrap up. 4. What are your general thoughts? If needed: How accurately do you feel the findings captured your thoughts/experiences? What do you think could be added to the findings to better capture your thoughts/experiences? If there is anything you would like removed, what would that be and why? 5. Is there anything else you’d like to tell me about yourself or the findings from the study?
